# Pyolaryngocele Presenting with Acute-onset Stridor

**DOI:** 10.5811/cpcem.57238

**Published:** 2023-09-29

**Authors:** Shyam Sabat, Luis Gonzalez, Amit Agarwal

**Affiliations:** * Mayo Clinic, Department of Radiology, Jacksonville, Florida; † University of Florida, Department of Radiology, Gainesville, Florida

**Keywords:** pyolaryngocele, stridor, computed tomography

## Abstract

**Case presentation:**

This case describes the classic imaging findings of pyolaryngocele and highlights the importance of prompt imaging for diagnosis of clinically occult airway lesions. The case also highlights how pyolaryngoceles can become large and present with acute-onset clinical symptoms, including stridor and dyspnea.

**Discussion:**

Pyolaryngoceles represent an uncommon but life-threatening complication of laryngoceles. Laryngoceles are frequently seen as an incidental, abnormal, air-filled dilation of the laryngeal saccule related to various local pathologies of the larynx. They are often asymptomatic. Occasionally they can become secondarily infected, in which case they are called pyolaryngocele, and they can cause rapid-onset, life-threatening airway compromise.

CPC-EM Capsule What do we already know about this clinical entity?

*Pyolaryngoceles are superinfected laryngoceles (8–10%) that present with muffled voice, odynophagia, and stridor.*

What is the major impact of the image(s)?

*Images show characteristic appearance of pyolaryngocele with smooth peripheral enhancement and central fluid contents.*

How might this improve emergency medicine practice?

*Clinicians should be aware of this entity and have a low threshold to recommend contrast-enhanced computed tomography for evaluation.*

## CASE PRESENTATION

A 61-year-old male presented to the emergency department (ED) with difficulty breathing, stridor, and fever (100°Farenheit) that developed over the course of 24 hours. Laboratory studies were significant for borderline leukocytosis with elevated neutrophil count. Contrast-enhanced computed tomography (CT) of the neck demonstrated a peripherally enhancing, lobulated fluid collection with layering debris within the right paraglottic space, with external extension through the right thyrohyoid membrane and severe airway compromise (Image).

**Image.**
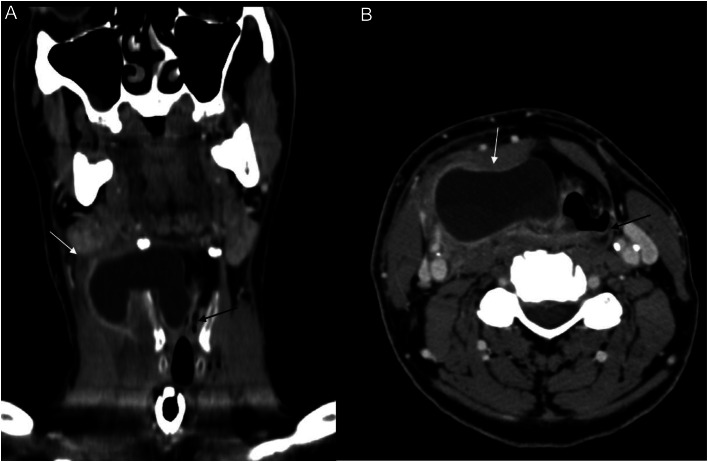
Coronal (A) and axial (B) contrast-enhanced computed tomography of the neck demonstrates a peripherally enhancing fluid collection arising from the right laryngeal vestibule, extending into the right anterior neck through the thyrohyoid membrane (*white arrow*), with narrowing of the laryngeal airway (*black arrow*), consistent with pyolaryngocele. Mild inflammatory changes noted in the surrounding soft tissues.

Imaging characteristics in conjunction with the clinical findings were consistent with pyolaryngocele. Emergency tracheostomy was performed to relieve the dyspnea, followed by surgical incision and drainage of the fluid collection, which was notable for pus. The patient was discharged in stable condition and was without complication at outpatient follow-up visit.

## DISCUSSION

Stridor is a high-pitched breathing sound produced by the abnormal flow of air, most prominently heard during inspiration. Although stridor is more common in children, it can present acutely across different age groups due to several conditions including foreign body aspiration, tracheitis, epiglottitis, and anaphylaxis. Abscesses along the pharyngeal and laryngeal compartments can also present with stridor, however, with a more subacute clinical course. Radiographic evaluation is usually the first line of investigation; however, this can frequently be unrevealing. Moreover, atypical clinical presentation and older age group should prompt the emergency physician toward further imaging work-up in the form of CT. Given the widespread availability of CT scanners in the ED, this is quickly replacing radiographs as the imaging modality of choice for evaluation of acute airway conditions.^1^

A laryngocele is an abnormal, air-filled dilation of the laryngeal saccule that communicates with the lumen of the larynx. It is believed to be secondary to increased laryngeal pressures and traditionally described in trumpet players; however, it is now more commonly seen secondary to excessive coughing and obstructive lesions.^2^ ^,^ ^3^ Laryngoceles can be classified into internal, external, or mixed (most common) subtypes based on whether or not the laryngocele is confined to the larynx or herniates through the thyrohyoid membrane.^4^ Laryngoceles are often asymptomatic but can become secondarily infected, in which case they are called pyolaryngoceles. These are rare clinical entities, presenting with fever, sore throat, and stridor. Imaging (CT) can identify the pyolaryngocele subtype, location, and extent of involvement of adjacent laryngeal structures, and aid in the treatment approach. Pyolaryngoceles are managed with drainage of the fluid collection and resection of the underlying laryngocele.^5^
